# Influence of Various Implant Placement Strategies on Stress Distribution in Maxillary Implant‐Retained Overdenture: A 3D Finite‐Element Analysis

**DOI:** 10.1002/cre2.70283

**Published:** 2026-01-09

**Authors:** Zahra Ghorbani, Ebrahim Shojaei, Hamid Neshandar Asli, Mehran Falahchai

**Affiliations:** ^1^ Department of Prosthodontics, Dental Sciences Research Center, School of Dentistry Guilan University of Medical Sciences Rasht Iran; ^2^ Helical Tosee Co. Guilan Iran

**Keywords:** finite‐element analysis, implant‐supported prosthesis, maxilla

## Abstract

**Objectives:**

This study used three‐dimensional (3D) finite‐element analysis (FEA) to investigate the effect of different implant placement strategies on the biomechanical behavior of implant‐supported maxillary overdentures, and provide an initial guide to clinical treatment.

**Materials and Methods:**

For an edentulous maxilla, six different implant‐supported overdenture models with various implant placement strategies were created using CATIA software. The reference model (5R‐3R‐3L‐5L) featured symmetrical implant placement in the canine and second premolar regions bilaterally. Five additional models incorporated asymmetrical implant placement strategies: 5R‐3R‐1L‐3L, 5R‐2R‐3L‐5L, 5R‐4R‐3L‐5L, 4R‐3R‐3L‐5L, and 6R‐3R‐3L‐5L. All models had identical bone properties, prosthetic components, material characteristics, and loading conditions. The geometric models were analyzed using ANSYS 24.0 Workbench software. The maximum principal stress for bone, and stress distribution patterns were analyzed, and the performance of the models was compared with the symmetrical reference model.

**Results:**

The quantitative and qualitative results showed that the implant placement strategy significantly influenced the magnitude and distribution of stress. The symmetrical implant placement strategy demonstrated the most favorable stress distribution, with the lowest maximum stress values in positions 5 R (2.69 MPa), 3 R (2.25 MPa), 3 L (2.16 MPa), and 5 L (3.24 MPa). Placement of implants in the anterior region resulted in stress concentration in the anterior region with maximum stress values at positions 5 R (3.24 MPa), 3 L (3.96 MPa), 1 L (5.09 MPa), and 3 L (5.57 MPa). Asymmetrical implant placement strategies with increased anteroposterior distribution and more posterior placement also demonstrated favorable biomechanical performance. Certain asymmetrical patterns induced fulcrum effects, leading to heterogeneous stress distribution.

**Conclusions:**

The symmetrical (5R‐3R‐3L‐5L) implant placement may provide a more uniform stress distribution, which may enhance peri‐implant bone preservation and long‐term implant stability. Implant placement in the canine region should be prioritized, while mesially‐positioned implants warrant clinical caution due to higher stress levels in bilaterally symmetrical implant placement strategies.

## 1 Introduction

Demographic transitions have led to a global rise in the aging population (Borg‐Bartolo et al. 2022), resulting in a higher prevalence of edentulism, which is a common oral health challenge among this age group (Tariq et al. 2024). The treatment options for edentulism include conventional complete dentures, implant‐retained overdentures (IODs), and implant‐supported full‐arch fixed prostheses (Kutkut et al. 2018). IODs offer a viable alternative that overcomes several limitations associated with conventional complete dentures in both the maxillary and mandibular arches. Studies have demonstrated that maxillary IODs provide better patient‐centered outcomes than conventional complete dentures, including improved functional capacity and less psychological, physical, and social discomfort (Zembic and Wismeijer 2014).

To improve the longevity of maxillary IODs, placement of a minimum of four implants is generally recommended (Di Francesco et al. 2019). A removable prosthesis supported by four implants and soft tissue in the maxilla is classified as an RP‐5 prosthesis. The pattern of load transfer from implants to the surrounding bone is strongly influenced by both the number and distribution of dental implants, underscoring the importance of strategic implant positioning (ELsyad et al. 2013). If implant positioning for a maxillary IOD is not properly planned at the beginning, it can seriously increase the risk of implant failure and prosthetic problems (Sadowsky and Zitzmann 2016). Given that overloading is a primary contributor to bone resorption around implants in RP‐5 overdentures (Yu et al. 2022), clinicians should be well aware of the pattern of stress and strain transmission to the peri‐implant bone when determining the optimal positioning of the four implants (Liu et al. 2013).

Finite element analysis (FEA) has become an accepted tool for analyzing the biomechanical behavior of structures (Wu et al. 2020), allowing for the estimation of stress distribution pattern and magnitude at different implant locations in an overdenture treatment plan. In maxillary IODs, FEA has been used to examine the impact of attachment type (Geramy and Habibzadeh 2018), bar attachment design, and palatal coverage (Kim and Hong 2016) on the biomechanical behavior of bone and prosthetic components. However, there is no specific evidence‐based protocol for the optimal arrangement of the four implants in RP‐5 prosthesis, and the biomechanical behavior of different implant placement strategies has rarely been analyzed by FEA (Di Francesco et al. 2019).

A recommended implant placement strategy for RP‐5, aiming to minimize premaxillary bone loss, involves clustering three implants anteriorly and one implant posteriorly in one side, resulting in an asymmetrical implant placement design (Misch 2004). However, no study has mathematically compared the magnitude and distribution of stress in bone surrounding the implants in this suggested configuration.

The exact position or anteroposterior distribution of the four implants has not been clearly defined in other studies that analyzed maxillary overdentures. The available evidence (Mañes Ferrer et al. 2020; Kappel et al. 2021; ELsyad et al. 2019) suggests that the anterior implants are usually placed in the canine region, while the posterior implants are symmetrically placed in the second premolar region (Liao et al. 2024). The area between the canines and second premolars is crucial for maximizing biomechanical advantage and mechanical stability (Assaf et al. 2022).

Assuming that bilateral implant placement in the canine and second premolar regions is commonly considered in the RP5 treatment plans, a perfect bilateral symmetry is rarely achieved. Biological and environmental factors such as premature tooth loss at one side, periapical cysts, traumatic extractions, asymmetrical sinus pneumatization, and poor bone quality at specific locations can influence decisions about symmetrical implant placement (Gönül et al. 1963; Damghani et al. 2012).

Despite the high incidence of such issues during treatment planning, no study has mathematically compared the magnitude and distribution of stress in the peri‐implant bone in various implant placement strategies or directly compared symmetrical versus asymmetrical implant placement configurations in maxillary overdentures. Therefore, this study investigated the effect of different implant placement strategies on stress distribution and biomechanical behavior of RP‐5 overdenture using FEA. This study aimed to enhance clinical outcomes by achieving favorable stress distribution in the overdenture assembly, minimizing stress concentration in bone, and increasing overdenture longevity. The research hypothesis suggested that bilateral implant placement in the canine and second premolar regions would provide a strategic and biomechanically favorable distribution, resulting in optimal stress reduction.

## 2 Materials and Methods

Evaluating the biomechanical response of complex treatment plans requires descriptive analysis of stress distribution patterns and numerical evaluation of stress within the bone‐implant‐denture system using FEA. In FEA studies, the assumptions related to geometry, mechanical properties of materials, applied loads, and boundary conditions play a critical role in determining the model accuracy (Saab et al. 2007).

**Figure 1:**
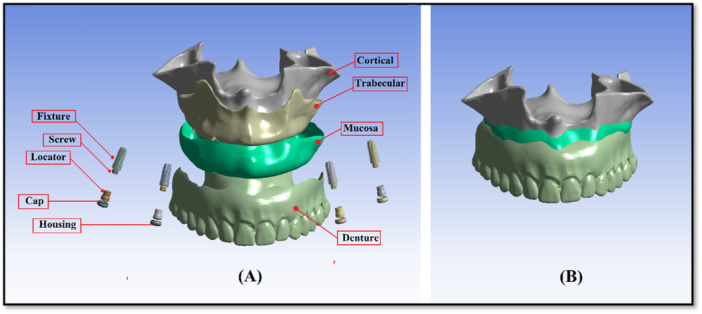
Model geometry development: (A) All individual components of the overdenture system are shown separately. Each component is displayed in its actual spatial orientation to illustrate the detailed geometry and interaction surfaces before assembly. (B) The finite element model after assembly, showing all components integrated into one single, cohesive system. This assembled model represents the final configuration used for finite element analysis.

**Figure 2:**
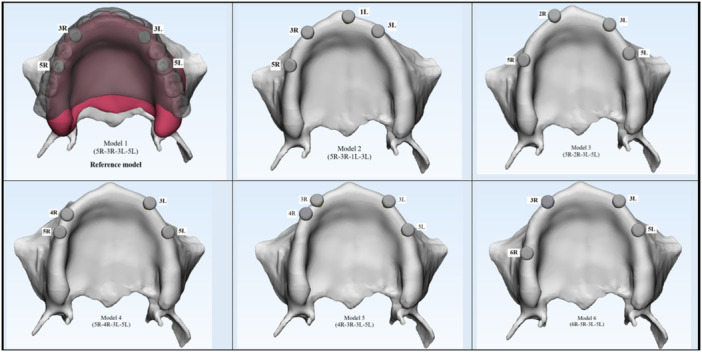
Schematic representation of six models of implant placement strategies.

**Figure 3:**
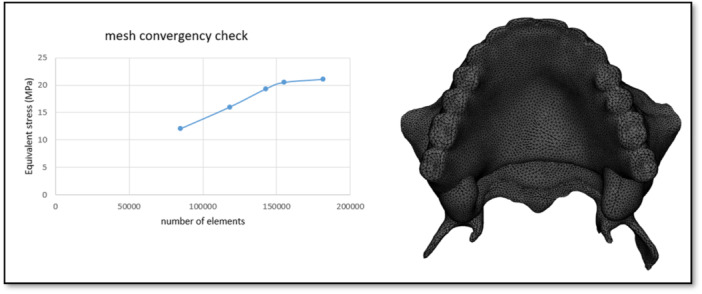
Mesh sensitivity test. This figure illustrates the results of the mesh sensitivity test conducted to ensure the accuracy and reliability of the finite element simulations. Different mesh densities were applied to the reference and asymmetrical models, and the effect on maximum principal stress values in the bone was evaluated. The analysis confirmed that a mesh configuration with a maximum of 5% error was sufficient to capture stress distribution patterns accurately, balancing computational efficiency and result precision. By performing this test, we ensured that the selected element size and node distribution did not significantly influence the simulation outcomes, providing confidence that the stress predictions reflect the true biomechanical behavior of the overdenture‐implant system.

In this study, a maxillary cone‐beam computed tomography scan (Aminianpour et al. 2025) of a 62‐year‐old edentulous patient was imported into Mimics software (Materialise Interactive Medical Image Control System; Materialise, Leuven, Belgium) to model a 3D geometry of the maxilla. This surface model was then transferred to CATIA software to construct a volumetric solid model. Based on the cone‐beam computed tomography scan, the maxillary cortical bone was modeled as one layer with 1.5 mm thickness, and the mucosa was modeled as one layer with 2 mm thickness. The individual implant components—including Straumann bone‐level tapered implants (4.2 × 10 mm; Institut Straumann AG, Basel, Switzerland) and locator abutments (Zest, USA)—were modeled using SolidWorks software (Dassault Systèmes). The denture, representing a custom‐made prosthesis for the edentulous patient, was scanned using a 3D optical scanner, and the scanned data were imported into CATIA software. The cortical bone, trabecular bone, mucosa, and IOD components, including dental implants, locator abutments, and prosthetic elements, were integrated into a unified model, maintaining anatomical fidelity (Figure 1). Six distinct RP‐5 models were created for a fully edentulous maxilla by using this reference model and altering the implant positions. The dome‐shaped top of the abutment was 2 mm above the alveolar ridge. A description of each model can be found in Figure 2 and Table 1. The solid models were transferred to ANSYS 24 software (ANSYS Inc., Canonsburg, PA), and the geometric models were converted into finite element mesh models using ANSYS 24.0 Workbench to generate the finite element mesh structures. Ten‐node quadratic tetrahedral elements were used for meshing. Mesh convergence was refined until the stress variation threshold fell below 5% (Figure 3). The corresponding number of elements and nodes for each model is reported in Table 2. In addition, the number of nodes and finite element elements in each component of the reference model is shown in Table 3.

**Table 1:** Description of the six finite‐element models with variable implant positions.

No.	Model name	Description
Model 1	(5R‐3R‐3L‐5L)	A fully symmetrical configuration was developed based on the primary study hypothesis to achieve optimal biomechanical behavior. Implants were placed bilaterally in the canine and second premolar regions (Figure 2A).
	Reference model	
Model 2	(5R‐3R‐1L‐3L)	An asymmetrical layout based on Professor Misch's recommendation to reduce premaxillary bone loss. Implants were placed in the right second premolar, right canine, left central incisor, and left canine regions (Figure 2B).
Model 3	(5R‐2R‐3L‐5L)	Simulating scenarios with anatomical constraints in the canine region on one side. Implants were positioned in the right second premolar, right lateral incisor, left canine, and left second premolar regions (Figure 2C).
Model 4	(5R‐4R‐3L‐5L)	Implants were placed in the right second and first premolars, left canine, and left second premolar regions. This model evaluates the biomechanical effect of mesial shifting on the right side (Figure 2D).
Model 5	(4R‐3R‐3L‐5L)	Designed to simulate limited implant placement in the right second premolar zone. Implants were placed in the right first premolar, right canine, left canine, and left second premolar regions (Figure 2E).
Model 6	(6R‐3R‐3L‐5L)	Featuring a posteriorly placed right implant to increase anteroposterior distribution. Implants were located in the right first molar, right canine, left canine, and left second premolar regions (Figure 2F).

**Table 2:** Number of finite‐element nodes and elements in each model.

Model (no.)	Elements (no.)	Nodes (no.)
1	304404	460638
2	305006	462972
3	305194	463104
4	304221	460537
5	305224	462994
6	304886	460367

**Table 3:** Number of finite‐element nodes and elements in each component of the reference model.

Reference model	Number of nodes	Number of elements
Cortical bone	69,426	37,864
Trabecular bone	79,370	43,406
Fixture	36,604	21,984
Gum	72,748	38,610
Screw	13,438	7644
Nylon	7230	4258
Housing	6952	3784
Attachment	6038	3410
denture	40,167	20,729
Total	331,973	181,689

The bone‐implant interface was defined as a fully bonded contact condition. This assumption represents a state of complete bone integration in which no relative movement occurs between the implant surface and the surrounding bone. Adopting a bonded contact surface is consistent with established FE modeling methods for bone‐bonded implants, and allows the simulation to represent the expected long‐term clinical conditions in which implant stability is achieved through direct bone contact without surface gaps or micromovements. This approach allows for accurate transmission of mechanical loads from the prosthetic components to the bone surrounding the implant, and avoids the generation of nonphysiological artifacts that would occur if slippage or separation were allowed at the contact surface. Therefore, the definition of bonded contact provides a realistic and acceptable representation of a fully‐bonded dental implant to bone within the framework of overdenture biomechanics.

**Figure 4:**
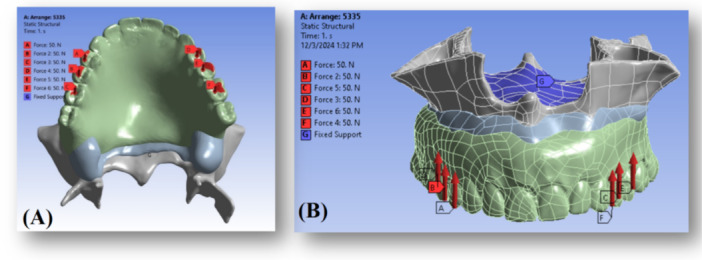
(A) An occlusal load of 300 N was applied bilaterally on the premolars and first molars (150 N per side) to simulate masticatory forces. (B) The surfaces constrained in all degrees of freedom are highlighted with blue dots, representing the fixed boundary conditions applied to the maxillary bone to prevent rigid body motion during simulation. These constraints ensure numerical stability and allow accurate calculation of stress distribution within the implant‐supported overdenture system.

To constrain the model and prevent rigid‐body motion, the superior surface of the maxilla was fully fixed. All translational degrees of freedom were also limited (blue dots in Figure 4B). These boundary conditions were chosen to replicate a physiologically relevant unilateral/bilateral occlusal loading scenario while preserving the mechanical role of the attachment friction and simulating a clinically realistic support by the cranial base.

Interaction between the locator attachment and the overdenture was modeled using the Coulomb friction law with a coefficient of friction μ = 0.30, which reflects the frictional retention mechanism of the metal housing and nylon insert.

The contact conditions between the denture and mucous membrane were defined as near frictionless (the coefficient of friction was considered to be 0.01 for denture‐mucosa to be assumed near frictionless) (Barão et al. 2013).

All components were assumed to be linearly elastic, homogeneous, and isotropic, following an elastic material constitutive model. The mechanical properties (elastic modulus and Poisson's ratio) assigned to each component are shown in Table 4.

**Table 4:** Material properties assigned in finite element analysis (Liao et al. 2024; Aminianpour et al. 2025; Khurana et al. 2019; Hong et al. 2012; Sezer et al. 2022; Eraslan et al. 2024).

Materials	Young's modulus (MPa)	Poisson's ratio
Cortical bone	13,700	0.30
Trabecular bone	1370	0.30
Mucosa	1	0.37
Titanium grade 4 (implant)	110,000	0.35
Polymethyl methacrylate (PMMA)	3000	0.35
Ti6Al4V (locator attachment and housing)	135,000	0.30
Nylon rubber	5	0.30

Although the amplitude of the load magnitudes differs across the literature, the maximum bite force has been determined to be a total of 300 N, with approximately 150 N per side, distributed over the first premolar, second premolar, and first molar teeth (Figure 4A), in agreement with previous research (Gümrükçü and Korkmaz 2018; Rismanchian et al. 2009). While the absolute load value is not essential due to the linearity of FEA, the linear nature of FEA means that the increase or decrease in stress levels is proportional to the applied load (Ferreira et al. 2014).

The von Mises equivalent stress distribution in the implants and the surrounding bone was selected as the primary descriptive comparison criterion. Additionally, the maximum principal stress (Pmax) values were used to quantitatively evaluate stress within the cortical and cancellous bones. For model evaluation, stress distribution was visualized using stress distribution contour maps, and stress values were reported in megapascals (MPa).

## 3 Results

**Figure 5:**
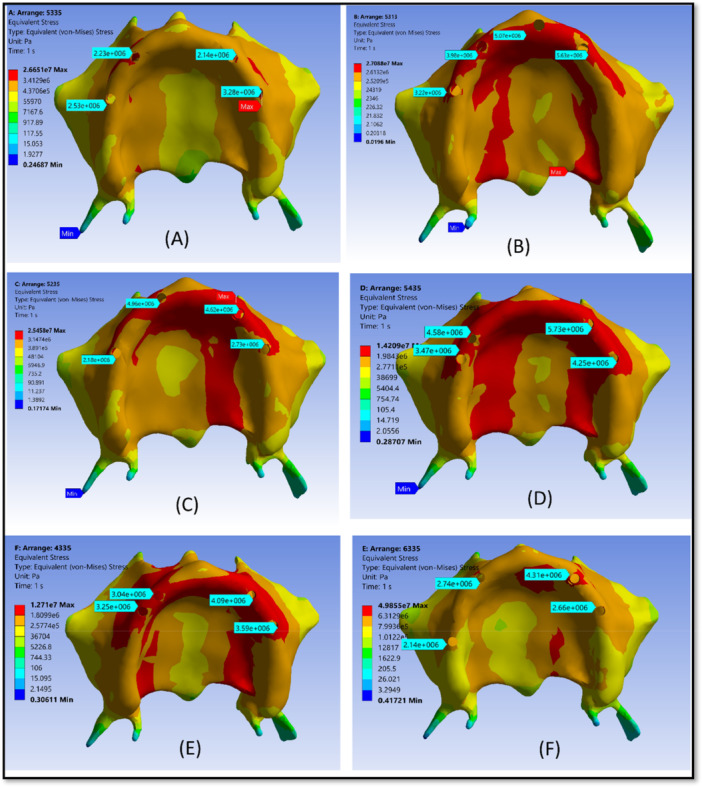
Stress distribution patterns in cortical bone across the six overdenture models based on the von Mises criterion. Each panel represents a different implant placement configuration: (A) Model 1 (5R–3R–3L–5L), (B) Model 2 (5R–3R–1L–3L), (C) Model 3 (5R–2R–3L–5L), (D) Model 4 (5R–4R–3L–5L), (E) Model 5 (4R–3R–3L–5L), and (F) Model 6 (6R–3R–3L–5L).The color scale indicates the magnitude of maximum principal stress in cortical bone, ranging from low stress (blue) to high stress (red). This qualitative visualization enables comparison of stress concentration patterns among the models. The reference model (A) exhibits a more uniform stress distribution, whereas certain asymmetrical configurations show localized stress peaks, highlighting areas at higher risk of overload and demonstrating the influence of implant position on biomechanical performance and bone preservation.

For all six models, the cortical bone stress distribution was visualized using color‐coded and quantitative scales (Figure 5); while the maximum von Mises stress of implants/prosthetic components and the maximum principal stress (Pmax) for bone (MPa) are summarized in Table 5.

**Table 5:** Quantitative comparison of stress distribution: maximum principal stress (Pmax) in bone, implants, and other prosthetic components.

Model no.	Implant position	Cortical bone stress (Pmax)	Cancellous bone stress (Pmax)	Implant neck stress (Pmax)	Locator cap stress (Pmax)
Model 1 (5R‐3R‐3L‐5L)	5 R	2.69 MPa	0.17 MPa	1.39 MPa	0.014 MPa
	3 R	2.25 MPa	0.27 MPa	1.12 MPa	9.89 e–3 MPa
	3 L	2.16 MPa	0.17 MPa	1.40 MPa	8.94 e–3 MPa
	5 L	3.24 MPa	0.24 MPa	1.83 MPa	0.01 MPa
Model 2 (5R‐3R‐1L‐3L)	5 R	3.24 MPa	0.34 MPa	2.15 MPa	0.012 MPa
	3 R	3.96 MPa	0.48 MPa	2.42 MPa	9.41 e–3 MPa
	1 L	5.09 MPa	0.52 MPa	2.17 MPa	0.010 MPa
	3 L	5.57 MPa	0.56 MPa	3.66 MPa	0.011 MPa
Model 3 (5R‐2R‐3L‐5L)	5 R	2.17 MPa	0.29 MPa	2.33 MPa	0.017 MPa
	2 R	4.89 MPa	0.48 MPa	3.32 MPa	0.014 MPa
	3 L	4.58 MPa	0.42 MPa	5.90 MPa	0.018 MPa
	5 L	2.56 MPa	0.26 MPa	3.62 MPa	0.023 MPa
Model 4 (5R‐4R‐3L‐5L)	5 R	3.66 MPa	0.37 MPa	2.89 MPa	0.016 MPa
	4 R	4.52 MPa	0.49 MPa	3.74 MPa	0.014 MPa
	3 L	5.49 MPa	0.56 MPa	4.73 MPa	0.015 MPa
	5 L	4.17 MPa	0.51 MPa	3.53 MPa	0.013 MPa
Model 5 (4R‐3R‐3L‐5L)	4 R	3.24 MPa	0.30 MPa	4.04 MPa	0.017 MPa
	3 R	3.08 MPa	0.34 MPa	3.32 MPa	0.012 MPa
	3 L	4.08 MPa	0.43 MPa	5.83 MPa	0.019 MPa
	5 L	3.51 MPa	0.35 MPa	4.98 MPa	0.017 MPa
Model 6 (6R‐3R‐3L‐5L)	6 R	2.13 MPa	0.28 MPa	1.12 MPa	0.014 MPa
	3 R	2.74 MPa	0.24 MPa	1.58 MPa	0.014 MPa
	3 L	4.31 MPa	0.69 MPa	3.82 MPa	0.016 MPa
	5 L	2.66 MPa	0.35 MPa	2.26 MPa	0.014 MPa

The cortical bone stress levels were consistently higher than those of the cancellous bone, regardless of the model and implant position. For instance, at the most distal implant site on the right side, the cortical bone stress varied across the six models, with values recorded at 2.69 MPa, 3.24 MPa, 2.17 MPa, 3.66 MPa, 3.24 MPa, and 2.13 MPa, respectively. At the same sites, stress values in the cancellous bone were 0.17 MPa, 0.34 MPa, 0.29 MPa, 0.37 MPa, 0.30 MPa, and 0.28 MPa, respectively. The ratio of cortical to cancellous bone stress reached a maximum of 13.5 in Model 1 (5R‐3R‐3L‐5L) and a minimum of 9.9 in Model 4 (5R‐4R‐3L‐5L).

In the reference model (5R‐3R‐3L‐5L), the maximum equivalent stress values in the cortical bone at the four implant sites were 2.69 MPa, 2.25 MPa, 2.16 MPa, and 3.24 MPa, respectively. However, Model 2 (5R‐3R‐1L‐3L) exhibited higher values at the corresponding sites: 3.24 MPa, 3.90 MPa, 5.09 MPa, and 5.57 MPa, respectively. The stress distribution pattern in the cortical bone, observed in both the reference model (5R‐3R‐3L‐5L) and Model 2 (5R‐3R‐1L‐3L), demonstrated that the reference model exhibited a more uniform stress distribution pattern.

The maximum equivalent cortical bone stress at implant sites in Model 3 (5R‐2R‐3L‐5L) and Model 4 (5R‐4R‐3L‐5L) was 2.17 MPa, 4.89 MPa, 4.58 MPa, and 2.56 MPa for Model 3, and 3.66 MPa, 4.52 MPa, 5.49 MPa, and 4.17 MPa for Model 4, respectively. The cortical bone stress at all implant positions in Model 4 demonstrated a marked increase compared to Model 3. Additionally, stress concentration increased markedly in Models 2, 3, and 4, relative to the reference model.

Comparing the stress distribution pattern and magnitude in the cortical bone between Models 5 and 6 revealed that Model 5 exhibited higher stress concentration than Model 6. In Model 5, the maximum equivalent cortical bone stress at the implant sites was 3.24 MPa, 3.08 MPa, 4.08 MPa, and 3.51 MPa, respectively; whereas, in Model 6, the corresponding values were 2.13 MPa, 2.74 MPa, 4.31 MPa, and 2.66 MPa, respectively.

## 4 Discussion

This study was performed to investigate the biomechanical behavior of different RP‐5 models with various implant placement strategies using FEA. An evaluation of the biomechanical properties of various implant placement strategies is essential to designing implant‐supported prostheses for optimal long‐term success (Yu et al. 2022). The results of this study confirmed the initial hypothesis that the (5R‐3R‐3L‐5L) model provides an optimal stress distribution pattern in RP‐5 overdenture. Stress analyses were performed on different prosthetic components, and stress levels in both trabecular and cortical bone were investigated.

Due to the intrinsic differences in bone properties, the cortical layer, which is characterized by a significantly higher Young's modulus relative to the cancellous core (Saab et al. 2007), absorbs the majority of stress. All models consistently demonstrated that the stress level in the cancellous bone was approximately 9–13 times lower than that in the cortical bone. This observation aligns with previous studies, including findings reported by Khurana et al. (2019), which further confirm that cortical bone primarily supports mechanical loads, regardless of implant arrangement.

The first modification to the reference model, aimed at evaluating the impact of altering implant position, was Model 2 (5R‐3R‐1L‐3L), which was an asymmetrical implant placement strategy recommended by Misch (2004) to prevent bone loss in the anterior maxilla. Based on numerical analysis, this placement strategy demonstrated suboptimal biomechanical performance. Concentrating three implants in the anterior region and removing the posterior implant on one side significantly increased stress concentration in the bone in the anterior region. Implant placement further anteriorly led to higher stress levels across all implants and resulted in a highly asymmetrical stress distribution pattern. Relative to the symmetrical reference model, this placement strategy resulted in a suboptimal stress distribution pattern.

However, the symmetrical model (5R‐3R‐3L‐5L), with implants placed bilaterally in the canine and second premolar regions, demonstrated optimal stress distribution across the peri‐implant bone. However, when anatomical limitations prevent this implant arrangement, alternative arrangements such as Models 3, 4, 5, or 6 may be considered. The changes in implant positions and their effects on both the magnitude and distribution of stress further emphasize the influence of implant placement on the biomechanical performance of RP‐5 overdentures.

In clinical scenarios where the 3 R position is not feasible, Models 3 (5R‐2R‐3L‐5L) and 4 (5R‐4R‐3L‐5L) offer alternative solutions. Moving the implant from the 3 R (canine) position to the 2 R (lateral incisor) position in Model 3 increased the cortical bone stress from 2.25 to 4.89 MPa, indicating a 117% rise. This displacement disrupted the stress distribution pattern and caused notable stress concentration. Similarly, in Model 4, moving the implant from the canine to the 4 R (first premolar) position increased the cortical bone stress from 2.25 to 4.52 MPa, indicating a 100% increase, and showing stress concentration due to asymmetry. These findings are consistent with those of Wu et al. (2020), who reported that in the “all‐on‐4” method, shifting anterior implants from the canine region to the incisors increased stress in the peri‐implant bone. Therefore, placing anterior implants in the canine region appears to facilitate mechanical balance between posterior and anterior implants, leading to a more favorable stress distribution pattern (Hussein and Rabie 2015). These findings indicate that when implant placement in the canine area is not feasible, any anterior or posterior displacement can negatively impact stress distribution.

In cases where placement in the 5 R position is not possible, the implant may be repositioned either more anteriorly (Model 5: 4R‐3R‐3L‐5L) or more posteriorly (Model 6: 6R‐3R‐3L‐5L). These changes reduce or increase the anteroposterior stress distribution, respectively. Among these, Model 6, with a posteriorly placed 6 R implant, demonstrated more favorable biomechanical performance in terms of both proportional stress distribution and stress magnitude.

As illustrated in Figure 5, stress distribution had a more uniform pattern in Model 6 (6R‐3R‐3L‐5L, Figure 5F) compared to Model 5 (4R‐3R‐3L‐5L, Figure 5E), without evident stress concentration at any implant site. These results align with the recommendations of Gümrükçü and Korkmaz [24] who emphasized that in asymmetrical implant placement strategies, posterior positioning helps minimize cantilever forces.

Regarding the magnitude of stress, in Model 5, where the replacement implant (5 R) was placed more anteriorly, the change in position led to an increase in stress concentration in the cortical bone surrounding all implants relative to the reference model. In Model 6, posterior repositioning of the right implant attenuated the cortical stress from 2.69 MPa to 2.13 MPa in the right side and from 3.24 MPa to 2.6 MPa in the left side, reflecting a 20.82% and 17.9% reduction, respectively, compared to the reference model. Comparing the torque arm, the distance between the last posterior implant and the distal end of the denture, revealed that Model 6 reduced the moment arm compared to the reference model (Model 1). This reduction corresponds to the increase in anteroposterior distribution in Model 6. Consequently, in the reference model (5R‐3R‐3L‐5L), during occlusal loading, when the posterior implant acts as a lever support, the distal extension of the denture resting on the soft mucosa functions as a free arm, doubling the pressure on the posterior implant. In Model 6, the shorter lever arm results in lower stress on the posterior implant and functions analogously to a first‐class lever theory. This result is consistent with the findings of ELsyad et al (ELsyad et al. 2013). However, this change in implant position in Model 6 resulted in da oubling of stress values in the implant located in the contralateral side at region 3. Notably, in Model 5, similar to Model 6, the highest increase in stress was observed in tooth position 3 of the contralateral side, showing an 88.9% rise, relative to the reference model. These findings indicate that in cases of asymmetrical placement of posterior implants on one side, stress is preferentially localized in the implant closest to the midline on the opposite side. This underscores the mechanical relevance of maintaining symmetry in implant placement across the arch, irrespective of posterior implant positioning. One explanation may be the anatomical location of the canine, which lies at the corner of the arch, potentially contributing to localized stress accumulation in the cortical bone of this region (Liao et al. 2024).

These findings indicate that in all asymmetrical models, the more mesially positioned implant is subjected to greater stress. Similarly, Liao et al. (2024) in their study on mandibular IODs found that, in clinical scenarios involving asymmetrical implant positions, the mesially positioned implant consistently experienced higher stress. Greater clinical vigilance is warranted for implants in these positions to prevent complications associated with uneven stress distribution. If asymmetrical placement of posterior implants on one side is unavoidable, it is advisable to use a wider‐diameter implant at the contralateral position 3 to increase load capacity.

Although this study is based on numerical FEA models, the observed stress distribution patterns may have important clinical implications. Models demonstrating lower cortical bone stress, such as the symmetrical 5R‐3R‐3L‐5L configuration, could potentially reduce the risk of marginal bone resorption and implant overload (Wu et al. 2020). Conversely, asymmetrical implant placement resulting in higher localized stress, particularly in mesially‐positioned implants, may cause harmful overloading of implant and overall peripheral bone structure leading to accelerated bone loss (Aboelfadl et al. 2024). These interpretations suggest that careful consideration of implant positioning, especially maintaining symmetry and optimizing anteroposterior distribution, may contribute to enhanced long‐term implant success.

This study was conducted to provide a general perspective on the consequences and effects of implant placement in different maxillary positions in RP‐5 overdenture on clinical biomechanical behavior. However, in order to focus on the implant placement variable, other influential factors—including arch shape, palatal overdenture coverage, intrinsic bone density variations, bone remodeling, and additional patient‐specific clinical variables—were not considered in this study. Furthermore, while FEA provides valuable biomechanical insights, several simplifying assumptions were applied, such as material homogeneity, linearly elastic behavior, 100% osseointegration, fixed boundary conditions, and static loading. In particular, the applied 300 N vertical occlusal load—although widely used as a standardized upper‐limit functional force in FEA studies—does not fully capture the true variability of clinical forces, which may differ by patient's age, muscular activity, bone quality, parafunctional habits, and occlusal scheme. Consequently, these factors prevent the direct translation of results to absolute clinical outcomes. The findings should therefore be interpreted as relative biomechanical comparisons among implant placement strategies. Future studies incorporating variable loading conditions, individualized anatomical models, dynamic forces, and long‐term bone remodeling effects are recommended to enhance clinical relevance and generalizability.

## 5 Conclusion

This study highlighted the biomechanical significance of the implant placement strategy in the biomechanical performance of the RP‐5 overdenture. The symmetrical implant placement strategies resulted in the best stress distribution patterns in maxillary overdenture treatment, while the asymmetrical implant configuration with three anterior implants led to localized stress accumulation in the bone in the anterior region. Overall, the findings highlight that symmetrical implant placement and optimized anteroposterior distribution in RP‐5 overdentures may support peri‐implant bone preservation and improve long‐term biomechanical performance.

When anatomical constraints prevent implant placement in position 5, posterior displacement is biomechanically preferable over anterior repositioning. The canine region should be prioritized for anterior implant placement to preserve optimal load transfer. Clinicians should exercise clinical vigilance when implants are placed more mesially, as this region is subjected to increased biomechanical loading in asymmetrical implant placement strategies.

## Author Contributions

**Zahra Ghorbani:** conceptualization, methodology, writing – original draft. **Ebrahim Shojaei:** Supervision, methodology, writing – review and editing. **Hamid Neshandar Asli:** investigation, resource. **Mehran Falahchai:** conceptualization, funding acquisition, project administration, writing – review and editing.

## Ethics Statement

This finite‐element analysis obtained ethical approval from the university ethics committee (IR. GUMS. REC.1403.297).

## Conflicts of Interest

The authors declare no conflicts of interest.

## Data Availability

The datasets used and/or analyzed in the current study are available from the corresponding author on reasonable request. Also, the datasets supporting the conclusions of this article are included within the article.
